# Chemotherapy-Related Amenorrhea and Menopause in Young Chinese Breast Cancer Patients: Analysis on Incidence, Risk Factors and Serum Hormone Profiles

**DOI:** 10.1371/journal.pone.0140842

**Published:** 2015-10-20

**Authors:** Giok S. Liem, Frankie K. F. Mo, Elizabeth Pang, Joyce J. S. Suen, Nelson L. S. Tang, Kun M. Lee, Claudia H. W. Yip, Wing H. Tam, Rita Ng, Jane Koh, Christopher C. H. Yip, Grace W. S. Kong, Winnie Yeo

**Affiliations:** 1 Department of Clinical Oncology and Comprehensive Cancer Trials Unit, Prince of Wales Hospital, The Chinese University of Hong Kong, Shatin, New Territories, Hong Kong SAR, China; 2 Department of Chemical Pathology and Li Ka Shing Institute of Health Science, Prince of Wales Hospital, The Chinese University of Hong Kong, Shatin, New Territories, Hong Kong SAR, China; 3 Department of Obstetrics and Gynaecology, Prince of Wales Hospital, The Chinese University of Hong Kong, Shatin, New Territories, Hong Kong SAR, China; School of Medicine, Fu Jen Catholic University, TAIWAN

## Abstract

**Purpose:**

In this prospective cross-sectional study on young premenopausal breast cancer patients, the objectives were to: determine the incidences of chemotherapy-related amenorrhea (CRA) and menopause (CRM); identify associated factors; and assess plasma levels of estradiol (E2) and follicular stimulating hormone (FSH) among patients who developed menopause.

**Methods:**

Eligibility criteria include Chinese stage I-III breast cancer patients, premenopausal, age ≤45 at breast cancer diagnosis, having received adjuvant chemotherapy, within 3–10 years after breast cancer diagnosis. Detailed menstrual history prior to and after adjuvant treatment was taken at study entry. Patients’ background demographics, tumor characteristics and anti-cancer treatments were collected. The rates of CRA and CRM were determined. Analysis was conducted to identify factors associated with CRM. For postmenopausal patients, levels of E2 and FSH were analyzed.

**Results:**

286 patients were recruited; the median time from breast cancer diagnosis to study entry was 5.0 years. 255 patients (91.1%) developed CRA. Of these, 66.7% regained menstruation. At the time of study entry, 137 (48.9%) had developed CRM, amongst whom 84 were age ≤45. On multivariate analysis, age was the only associated factor. Among patients with CRM, the median FSH was 41.0 IU/L; this was significantly lower in those who were taking tamoxifen compared to those who were not (20.1 vs. 59.7 IU/L, p<0.0001). The E2 level was <40 pmol/L; there was no difference between those who were still on tamoxifen or not.

**Conclusion:**

After adjuvant chemotherapy, the majority of young Chinese breast cancer patients developed CRA; ~50% developed CRM, with 61% at age ≤45. Age at diagnosis is the only factor associated with CRM. FSH level may be affected by tamoxifen intake.

## Introduction

Over the past two decades, breast cancer has become the most common female cancer in Hong Kong, and in 2012, there were over 3500 new cases [1]. Over 80% of newly diagnosed breast cancer patients in Hong Kong have early stage disease [1], and in a recent report, of the 7152 patients entered into the Hong Kong Breast Cancer Registry, about 14% of the patients were younger than 40 at diagnosis [2]. Women with early stage breast are indicated for curative treatments which include surgery followed by adjuvant chemotherapy, radiation therapy and hormonal, and based on recent report, younger breast cancer patients have a higher proportion having received cytotoxic chemotherapy as part of the adjuvant treatments [2]. Anticancer treatment, especially cytotoxic chemotherapy, is associated with immediate as well as long-term toxicities; the latter include transient period of amenorrhea as well as the potential of permanent amenorrhea and early menopause, which may affect the quality of life and well-being of cancer survivors [3–8].

Most of the studies on long-term chemotherapy-related toxicities in younger breast cancer survivors have been done in the West wtih limited data based on Asian patients [3–13]. For studies on chemotherapy-related amenorrhea (CRA), interpretation of available data is hindered by the fact that different definitions of CRA have been used, with amenorrhea period varying from 3 months to 12 months. Further, there has been limited data on detailed menstrual history after CRA (21–24). Specifically, the delineation between CRA, the resumption of menses after a period of CRA, and the proportion of women who subsequently develop menopause (in particular, early menopause) has not been well described. Moreover, incidence of early menopause has also not been well described. Such information is of particular interest in the light of available additional adjuvant endocrine therapy with aromastase inhibitors and gonadotropin releasing hormone (GnRH) agonists. For instance, the NCCN guidelines recommend that when considering adjuvant endocrine therapy with an aromatase inhibitor, women age 60 year or younger taking tamoxifen (or toremifene) should have follicle stimulating hormone (FSH) and plasma estradiol (E2) levels in postmenopausal ranges [14]. However, the exact timing of these blood tests and the levels of FSH and E2 have not been well defined.

In this prospective cross-sectional study on young Chinese premenopausal with early breast cancer in Hong Kong, the objectives were (1) to determine the incidence of chemotherapy-related amenorrhea (CRA) and menopause (CRM), (2) to identify the associated risk factors with CRM, and (3) among patients who developed CRM, to assess the plasma levels of FSH and E2.

## Patients and Methods

Between September 2008 and February 2011, 286 patients were enrolled into this study. Eligibility criteria included female of Chinese ethnicity, stage I-III breast cancer, premenopausal and age younger than 45 years at breast cancer diagnosis, having received adjuvant chemotherapy, within 3–10 years after breast cancer diagnosis at recruitment. Patients who received ovarian ablation as part of the endocrine therapy and patients who had hysterectomy prior to breast cancer diagnosis were excluded from the study. Patients who came for their follow up visit at the Prince of Wales Hospital were consented for the study. After written consent, they were asked to fill in a study questionnaire in which they recalled their menstruation history with the assistance of a research nurse. Patients’ demographics and details of their breast cancer characteristics and anti-cancer treatment at diagnosis were recorded. Individual’s hormonal profile based on serum estradiol (E2) and follicle stimulating hormone (FSH) were determined where indicated (see below). The study was approved by the Joint CUHK-NTEC Clinical Research Ethics Committee of the Chinese University of Hong Kong and Hong Kong Hospital Authority.

### Menstrual history since breast cancer treatments and definition of menopausal status

Patients were asked in details the following: [1] last menstrual period (LMP) before commencement of chemotherapy; [2] after commencement of chemotherapy, presence or absence of a period of amenorrhea with dates; [3] in case of occurrence of amenorrhea, whether there was subsequent return of menstruation with dates; [4] for patients who had resumption of menstruation, any subsequent amenorrhea with dates. For patients who did not experience amenorrhea during chemotherapy, the subsequent menstrual history was further collected similarly. LMP prior to study entry was determined for each patient.

Chemotherapy-related amenorrhea (CRA) was defined as amenorrhea for ≥3 months during and within 12 months after the completion of adjuvant chemotherapy. Return of menstruation was defined as at least 2 cyclical menstrual bleeding after CRA.

Chemotherapy-related menopause (CRM) was defined in line with WHO criteria as 12 months of amenorrhea with LMP ≥12 months after chemotherapy and before study entry [15]. Premenopausal status was defined as menstruation at least every 3 months with LMP within 3 months prior to study entry.

For patients determined to be have developed CRM, the ‘age of menopause’ was determined from the time of LMP. ‘Early menopause’ was defined as having reached CRM at the age of 45 or younger.

### Measurement of E2 and FSH

For patients who were determined to be menopause, serum FSH and E2 were measured by electrochemiluminescence immunoassay with Roche commercial kits on Cobas E601 Analyser. The lower detection limit of E2 was 18.4 pmol/L. Measurement imprecisions were less than 10% coefficient of variation.

### Statistical Analysis

Patients who developed menopause were compared with those who did not in an attempt to identify potential factors in association with menopause.

Statistical analysis was performed by SAS version 9.3. Continuous variables were expressed as means with standard deviation or median with range as appropriate. Baseline continuous variables were compared by Student’s t-test or Mann Whitney U test as appropriate, and categorical variables were compared by Chi’s square test. All statistical tests were two-sided, and p values less than 0.05 were regarded as significant.

Univariate Logistic regression will be performed to identify any prognostic factors associated with menopause. Stepwise multivariate logistic regression analysis included significant factors was conducted.

Among patients who developed CRM, analysis was conducted on serum levels of FSH and E2. The criteria adopted for postmenopausal level of FSH was >40 IU/L and that of E2 was <44 pmol/L [16].

## Results

A total of 286 breast cancer patients were consented to participate in this study. Two patients failed to meet inclusion criteria as they received neo-adjuvant therapy for their stage IIIb breast cancers, 4 patients withdrew with the reason that they didn’t have time to perform the blood tests after consent. As a result, 280 patients entered the study. Table 1 shows the patients’ background demographics and clinical characteristics, tumor characteristics, breast cancer treatments received at breast cancer diagnosis. The clinical information database is shown in S1 Table.

**Table 1:** Patients’ background demographic and clinical characteristics at the time of breast cancer diagnosis.

	No. of patients	%
Age at diagnosis		
≤35	41	14.6
36–40	82	29.3
41–45	157	56.1
BMI at diagnosis (according to HK BMI)		
Underweight (<18.5) to Normal (18.5–22.9)	188	67.2
Overweight (23.0–24.9) to Obese (>25)	92	32.8
Education		
Primary school	47	16.9
Secondary school	187	67.0
Tertiary school	27	9.7
Higher qualification	18	6.5
Height- median; range (cm)	159 (141–175)	
Weight- median; range (kg)	54.6 (39.0–89.0)	
Number of children born before breast cancer diagnosis		
0	75	26.8
1 or more	205	73.2
Smoking		
Yes	10	3.6
No	270	96.4
Alcohol (Excessive (> 2 units / day) alcohol intake:)		
Yes	1	0.4
No	279	99.6
1^st^ degree relative with breast cancer	17	6.1
T stage		
T1	140	50.0
T2	133	47.5
T3	7	2.5
T4	0	0
Nodal Status		
Positive	114	40.7
Negative	166	59.3
TNM staging:		
Stage I	88	31.4
Stage II	165	58.9
Stage IIIa	27	9.6
Hormonal receptor		
ER positive	203	72.5
PR positive	187	66.8
ERB-2 neu		
Over expression	47	16.8
No over expression	233	83.2
Breast surgery:		
Lumpectomy	95	33.9
Mastectomy^1^	185	66.1
Axillary Lymph Node dissection		
ALND	276	98.6
No ALND	4	1.4
Adjuvant Radiotherapy		
No radiotherapy	94	33.6
Locoregional radiotherapy (LRRT)	92	32.8
Breast / Chest wall	94	33.6
Chemotherapy regimen^2^:		
AC x 4	153	54.6
CMF x 6	15	5.4
AC x 4 followed by CMF x 3	28	10
AC x 4 followed by T x 4	68	24.3
Others	16	5.7
Duration of chemotherapy		
< = 64 days	89	31.8
> 64 days	191	68.2
Use of corticosteroid during chemotherapy premedication		
Yes	258	92.1
No	22	7.9
Hormonal therapy: Tamoxifen		
Yes	214	76.4
No	66	22.6
Trastuzumab		
Yes	8	2.9
No	272	97.1
Use of traditional Chinese medicine since diagnosis		
Yes	83	29.6
No	197	70.4
Having experienced chemotherapy-related amenorrhea	255	91.1

^1^ 25 patients had mastectomy with breast reconstruction

^2^ AC- doxorubicin+cyclophosphamide; CMF- cyclophosphamide+methotrexate+fluorouracil; T- paclitaxel, numbers indicated number of cycles received; ‘Others’ included 8 patients who had anthracycline-taxane containing regimens other than AC-T, 4 who did not complete CMF x 6, 1 who did not complete AC x 4, 1 who had paclitaxel x 4 only, 1 who had docetaxel+ cyclophosphamide x 4 and 1 who had CMF x 1 and paclitaxel x 4.

The BMI results were divided into four categories according to WHO criteria [17].

Of the 280 patients recruited, the median age at breast cancer diagnosis was 41 years (range: 24–45); at the time of breast cancer diagnosis, 41 patients were ≤35 years, 82 were aged 36–40 and 157 were aged 41–45. The median age at study entry was 46.5 years (range: 28–54); 8 were ≤35 years, 26 were aged 36–40, 76 were aged 41–45, 146 were aged 46–50 and 24 were aged >50. According to the BMI for Asians population [17], 11.8% were underweight, 55.4% were normal, 16.54% were overweight, and another 16.4% were obese. The median time from breast cancer diagnosis to study entry was 5.04 years (range: 2.96–9.94). Eighty-eight had Stage I, 165 had stage II and 27 had Stage III breast cancer. Adjuvant chemotherapy regimens included anthracycline-containing (64.6%; doxorubicin+cyclophosphamide [AC] with or without cyclophosphamide+methotrexate+fluorouracil [CMF]), anthracycline-taxane containing (24.3%), others (11.1%; including CMF alone).Two hundred and fourteen patients also received adjuvant tamoxifen; at the time of the study, 99 patients had completed tamoxifen and while 115 patients were still on adjuvant tamoxifen therapy. No patient received adjuvant aromatase inhibitors.

### Incidence of chemotherapy-related amenorrhea and menopause

**Fig 1:**
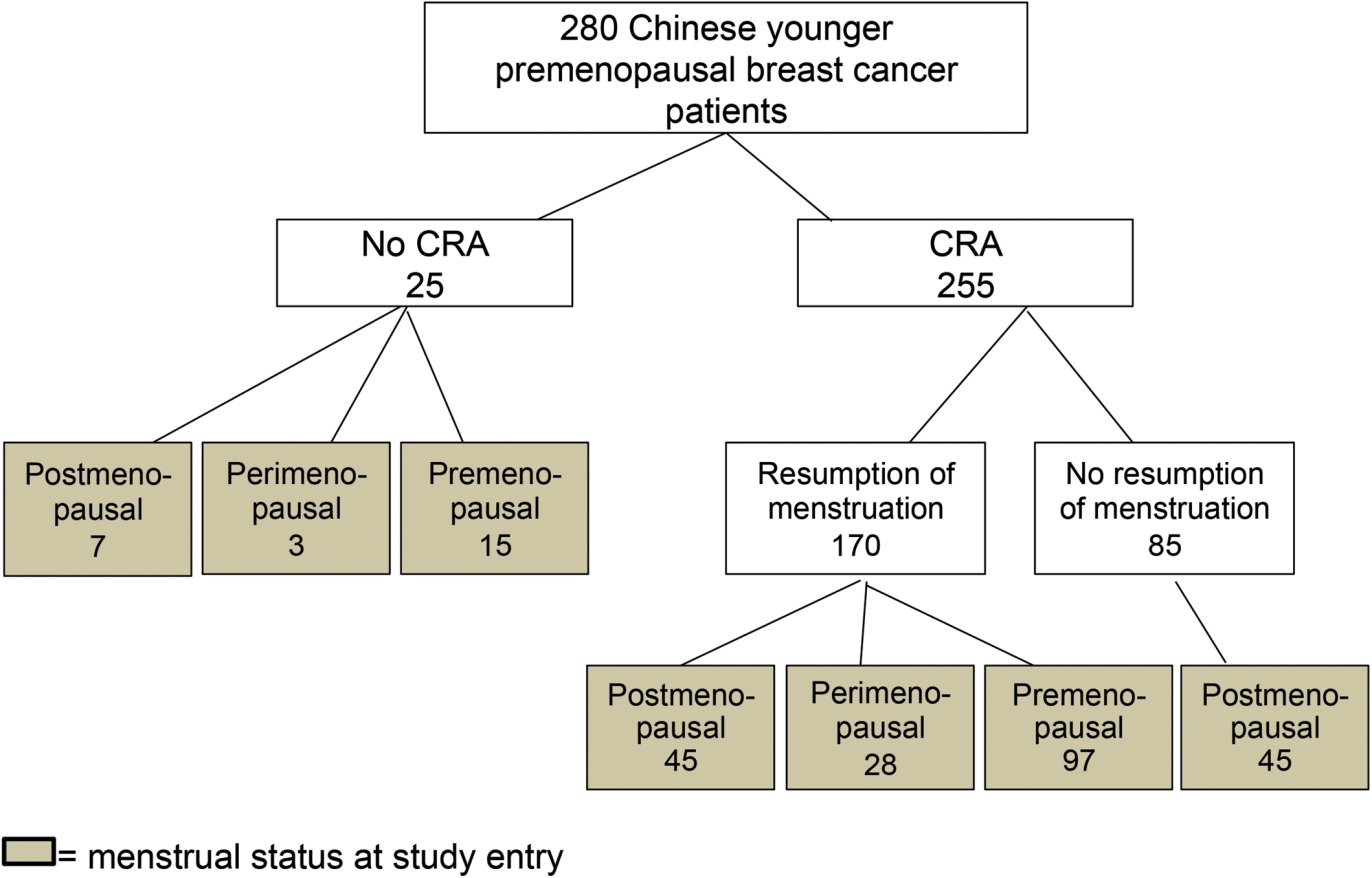
The menstrual status of studied population after receiving adjuvant chemotherapy.

Of the 280 patients, 255 (91.1%) developed CRA (Fig 1). Twenty-five patients continued menstruation during and within 12 months after completion of adjuvant chemotherapy; 9 had regular and 16 had irregular menstrual cycles. At study entry, 7 of these 25 had developed menopause.

Of the 255 who had CRA, 170 (66.7%) resumed their menstruation; the median period of CRA was 8.95 months (range: 3.0–75.2). Among the 170 patients who resumed menstruation, 28 (16.5%) regained menses within 6 months of completion of chemotherapy, 83 (48.8%) regained menses between 6–12 months, and 59 (34.7%) regained menses more than 12 months after completion of chemotherapy. At study entry, 45 of the 170 patients had developed menopause, 97 remained to be premenopausal and 28 were considered to be peri-menopausal.

Therefore, at the end of the study, a total of 137 patients (including 45 who experienced CRA and 7 who did not experience CRA) had CRM (48.9%). The median age at CRM was 44 years (range: 34–52); the median interval from last menstrual period to study entry was 4.3 years (range: 1.0–9.8). Of the 137 patients who developed CRM, 84 (61.3%) had early menopause (i.e. at age ≤45).

Of those who remained pre-menopausal, there was no live birth after breast cancer diagnosis and treatments.

### Analysis for risk factors associated with the development of early menopause

Table 2 compares the background characteristics of patients who did and did not develop CRM.

**Table 2:** Univariate analysis on factors associated with chemotherapy-related menopause.

	No. of patients (%)		
	Patients with menopause (n = 137)	Patients without menopause (n = 143)	P
Age at diagnosis			<0.0001
≤35	4 (2.9)	37 (25.8)	
36–40	29 (21.2)	53 (37.1)	
41–45	104 (75.9)	53 (37.1)	
BMI at diagnosis (according to HK BMI)			0.0754
Underweight (<18.5) to Normal (18.5–22.9)	85 (62.0)	103 (72.0)	
Overweight (23.0–24.9) to Obese (>25)	52 (38.0)	40 (28.0)	
Education			0.0167
Primary school	31 (22.8)	16 (11.2)	
Secondary school	87 (64.0)	100 (69.9)	
Tertiary school	8 (5.9)	19 (13.3)	
Higher qualification	10 (7.3)	8 (5.6)	
Height- median; range (cm)	158 (146–170)	159 (141–175)	0.9389
Weight- median; range (kg)	55.0 (39.0–88.5)	54.0 (40.0–89.0)	0.9639
Number of children born before breast cancer diagnosis			0.0762
0	32 (23.4)	43 (30.1)	
1 or more	105 (76.6)	100 (69.9)	
Smoking			0.9450
Yes	5 (3.7)	5 (3.5)	
No	132 (96.3)	138 (96.5)	
Alcohol (Excessive (> 2 units / day) alcohol intake:)			0.4893
Yes	1 (0.7)	0	
No	136 (99.3)	143 (100)	
1^st^ degree relative with breast cancer	10 (7.3)	7 (4.9)	0.3997
Hormonal receptor			
ER positive	101 (73.7)	102 (71.3)	0.6538
PR positive	89 (65.0)	98 (68.5)	0.5263
ERB-2 neu			0.7482
Over expression	24 (17.5)	23 (16.1)	
No over expression	113 (82.5)	120 (83.9)	
Adjuvant Radiotherapy			0.5752
No radiotherapy	49 (35.8)	45 (31.5)	
Locoregional radiotherapy (LRRT)	46 (33.6)	46 (32.2)	
Breast / Chest wall	42 (30.7)	52 (36.4)	
Chemotherapy regimen^2^:			0.0341
AC x 4	72 (52.5)	81 (56.6)	
CMF x 6	10 (7.3)	5 (3.5)	
AC x 4 followed by CMF x 3	20 (14.6)	8 (5.6)	
AC x 4 followed by T x 4	30 (21.9)	38 (26.6)	
Others	5 (3.7)	11 (7.7)	
Duration of chemotherapy			0.3625
< = 64 days	40 (29.2)	49 (34.3)	
> 64 days	97 (70.8)	94 (65.7)	
Use of corticosteroid during chemotherapy premedication			0.5830
Yes	125 (91.2)	133 (93.0)	
No	12 (8.8)	10 (7.0)	
Hormonal therapy: Tamoxifen			0.9343
Yes	105 (76.6)	109 (76.2)	
No	32 (23.4)	34 (23.8)	
Trastuzumab			0.1174
Yes	2 (1.5)	6 (4.2)	
No	135 (98.5)	137 (95.8)	
Use of traditional Chinese medicine since diagnosis			0.9188
Yes	41 (29.9)	42 (29.4)	
No	96 (70.1)	101 (70.6)	
Having experienced chemotherapy-related amenorrhea			
Yes	131 (95.6)	126 (88.1)	0.0222
No	6 (4.4)	17 (11.9)	

On univariate analysis (Tables 2 and 3), older age at breast cancer diagnosis (OR 3.938, 95% CI 2.606–5.949; p<0.0001) and having experienced CRA were associated with CRM (OR 2.674, 95% CI 1.080–6.623, p = 0.0335). There was a trend that higher BMI (i.e. overweight or obese) (OR 1.575, 95% CI 0.953–2.603, p = 0.0762) and difference in educational level (OR 0.738, 95% CI 0.528–1.032, p = 0.0754) being associated with CRM. On multivariate analysis (Table 3), age was the only factor associated with CRM; at breast cancer diagnosis, patients who were age 36–40 had an increased risk of 5.061 when compared with those age 35 or less (95% CI 1.641–15.614, p = 0.0048), and those age 41–45 had an increased risk of 18.151 when compared with those age 35 or less (95% CI 6.143–53.626, p<0.0001).

**Table 3:** Univariate and Multivariate analysis on factors associated with chemotherapy-related menopause by stepwise logistic regression.

	Univariate			Multivariate analysis		
	Odd Ratio (OR)	95% CI for OR	p	Odd Ratio (OR)	95% CI for OR	p
Age at diagnosis	3.938	2.606–5.949	<0.0001	3.938	2.606–5.949	<0.0001
≤35	1	-	-	1	-	-
36–40	5.061	1.641–15.614	0.0048	5.061	1.641–15.614	0.0048
41–45	18.151	6.143–53.626	<0.0001	18.151	6.143–53.626	<0.0001
BMI at diagnosis (according to HK BMI)	1.575	0.953–2.603	0.0762			
Underweight (<18.5) to Normal (18.5–22.9)						
Overweight (23.0–24.9) to Obese (>25)						
Education	0.738	0.528–1.032	0.0754			
Primary school	1	-	-			
Secondary school	0.435	0.224–0.846	0.0142			
Tertiary school	0.211	0.076–0.585	0.0028			
Higher qualification	0.625	0.207–1.890	0.4052			
Height- median; range (cm)	0.998	0.959–1.040	0.9386			
Weight- median; range (kg)	0.999	0.974–1.026	0.9638			
Number of children born before breast cancer diagnosis	1.333	1.006–1.767	0.0451			
0						
1 or more						
Smoking	1.045	0.296–3.694	0.9450			
Yes						
No						
Alcohol (Excessive (> 2 units / day) alcohol intake:)	-	-	0.9856			
Yes						
No						
1^st^ degree relative with breast cancer	1.530	0.565–4.141	0.4027			
Hormonal receptor						
ER positive	1.128	0.667–1.907	0.6542			
PR positive	0.851	0.518–1.401	0.5265			
ERB-2 neu	1.108	0.592–2.074	0.7482			
Over expression						
No over expression						
Adjuvant Radiotherapy	0.861	0.647–1.147	0.3076			
No radiotherapy	1	-	-			
Locoregional radiotherapy (LRRT)	0.918	0.517–1.632	0.7717			
Breast / Chest wall	0.742	0.418–1.316	0.3074			
Chemotherapy regimen^2^:	0.961	0.817–1.130	0.6277			
AC x 4						
CMF x 6						
AC x 4 followed by CMF x 3						
AC x 4 followed by T x 4						
Others						
Duration of chemotherapy	1.264	0.763–2.094	0.3629			
< = 64 days						
> 64 days						
Use of corticosteroid during chemotherapy premedication	0.784	0.327–1.878	0.5843			
Yes						
No						
Hormonal therapy: Tamoxifen	1.024	0.589–1.778	0.9343			
Yes						
No						
Trastuzumab	0.338	0.067–1.706	0.1891			
Yes						
No						
Use of traditional Chinese medicine since diagnosis	1.027	0.615–1.716	0.9188			
Yes						
No						
Having experienced chemotherapy-related amenorrhea	0.339	0.130–0.889	0.0278			
Yes						
No						

### Analysis on serum levels of FSH and E2

Among the patients who developed CRM, the median level of FSH was 41.0 IU/L (range: 6.3–144). One hundred and two patients received tamoxifen as part of their adjuvant therapy; of these 52 were still on tamoxifen at the time of the study. The median level of FSH for those who were on tamoxifen was 20.1 IU/L (range: 6.3–71.5); this was significantly lower than that of those who had completed tamoxifen therapy, in whom the corresponding level was 59.7 IU/L (range: 10.8–119.0) (p<0.0001).

The median level of E2 was <44 pmol/L (range: <44–>3000); 104 patients (75.9%) had E2 level <44 pmol/L. The median level of E2 for those who were on tamoxifen as well as those who had completed tamoxifen therapy were both <44 pmol/L (p = 0.7973).

## Discussion

In the past decades, oncologists have mainly focused on survival and early detection of cancer recurrence during follow-up visits of surviving patients. With the improvement in breast cancer treatments, cancer survivors now have longer survivals. Long-term toxicities associated with cancer treatments become more evident with increase survival, and these include effects on physical morbidities and psychosocial symptoms. In this study, we have focused on chemotherapy-related menstrual disturbances in young breast cancer survivors. In contrast to previous studies to address this area, we have paid specific attentions to delineate CRA (which may be associated with return of menses), and menopause (which indicates permanent loss of menstruation).

Our study showed that among young Chinese breast cancer patients who underwent adjuvant chemotherapy, 91% experienced CRA, 49% developed CRM amongst whom 61% had early menopause. In consistent with previous studies, age was an important factor for CRM after adjuvant chemotherapy [18–20], and having experienced CRA has been reported to have an increased risk of menopause compared to those who continue to menstruate throughout chemotherapy [3].

The exact mechanisms that cytotoxic chemotherapy causes disruption in menstrual cycles remain unknown. Alkylating agents (such as cyclophosphamide), cell cycle inhibitors (such as anthracyclines) and antimetabolites (such as methotrexate and fluorouracil) affect stability of quiescent and growing follicles leading to the disruption of normal menstrual cycles and CRA [16,21,22]. The risk of amenorrhea also increases with higher dose of chemotherapy, and when anthracyclines, cyclophosphamide and taxanes are used in combination [22]. For patients who had undergone chemotherapy, some studies have shown that the addition of tamoxifen substantially affected the onset of menopause [11,23–24], while other studies have not [25–26]. In the current study, the significance of individual agents and the duration of chemotherapy on menstrual disturbances are difficult to be ascertained due to limited number of patients studied; further, all patients received alkylating agent, with most of the described agents being used in combination regimens, while over three-quarters also had tamoxifen.

In a recent report among Chinese women, sociodemographic factors were analyzed in association with age at menopause. Factors identified to be associated with early menopause include current smokers, current alcohol drinkers, unmarried status and lower household income; while having attained a high school education was associated with older age at menopause [27]. Interestingly, another study on Iranian women reported that those with higher socioeconomic level experience later menopause [28]. In the present study, although not statistically significant, a higher proportion of patients who had higher education level did not experience CRM. Cigarette smoking has been identified to be a strong predictor of early menopause in studies of natural menopause [29–30], and this has been linked to benzopyrene and other polycyclic aromatic hydrocarbons causing destruction of primordial follicles in animal models [31]. In patients undergoing chemotherapy, it has also been suggested that smokers have greater risk of CRA when compared to never smokers [32]. The effect of smoking is difficult to be ascertained within the current study, as only 3.6% of our patient population was ever smokers. For the same reason, the effect of alcohol cannot be ascertained in the present study, as only one out of the total of 280 studied patients admitted to excessive alcohol intake of over 2 units per day.

There have been inconsistent findings on the association of weight or BMI with timing of natural menopause. In women who have undergone chemotherapy, Powis et al reported that obese patients have decreased clearance of cyclophosphamide, which could result in the development of CRA [33]. While some studies observed no association [13,34], others have reported a delayed [30,31,35], or the contrary of earlier onset menopause [36] in obese women. The current study has not been able to confirm an association of BMI with CRM.

The present study has a few limitations. One being related to the cross-sectional design with no long term follow-up, and patients were asked to recall their menstrual history which may create bias. Further, hormone profiles were only determined once, which would be considered by many to be inadequate in determining menopausal status. However, although serial monitoring of hormone profiles has been recommended, the timing, duration and criteria for defining menopausal status after chemotherapy have not been well established [37–38].

The complexity of ovarian change after chemotherapy makes it difficult to determine when hormone measurement should be performed after a period of amenorrhea to confirm postmenopausal status. Although, some studies suggested that the sensitivity of the plasma E2 immunoassay might not be sufficiently sensitive to detect the low postmenopausal E2 levels [39], newer assays like the one used in this study have shown very good sensitivity in confirming biochemical ovarian failure among CRM patients. Patients who are on tamoxifen have been reported to have increased E2 while decreased FSH levels, and this has been partly attributed to cross-reactivity of tamoxifen and its metabolites in the estradiol assay [40, 41]. In the current study, the median estradiol level of menopausal patients was <44 pmol/L; this did not differ between patients who were still on adjuvant tamoxifen therapy or not. On the contrary, FSH was found to be significantly lower amongst those who were still on tamoxifen, and this could have been due to tamoxifen interfering with the normal negative pituitary feedback mechanisms [42–43].

Other than tamoxifen, there are other potential interference substances in E2 assays, such as herbal medicine. The utilization of traditional Chinese medicine (TCM) is a common practice in Chinese population as it is believed to alleviate chemotherapy-related toxicities and improve well-being on one hand, while exert anti-cancer effect on the other. This is evident in the current study, which shows that about 30% of the studied patients adopted the use of TCM, with some of such concoctions potentially containing estrogen-like compounds [44]. The latter is demonstrated in one menopausal patient in the current study who was detected to have an estradiol level that exceeded 3000 pmol/L while she was taking TCM; upon discontinuation of the TCM, the estradiol level decreased to <44 pmol/ml.

More recently, anti-Müllerian hormone (AMH) and inhibin B have been shown to improve the predictive capacity of ovarian function and reflect subtle changes in menstrual transition when compared with FSH, and they appear not to be affected by the administration of tamoxifen [45–46]. In addition, antral follicle count (AFC) has also been suggested to be a useful parameter among children cancer survivors, while reduced AFC was observed in breast cancer patients after chemotherapy and appeared to be in accordance with suppressed ovarian function [41,47]. However, studies on AMH and inhibin B are so far limited by the small patient number with inhomogeneity in age distribution, chemotherapy regimen and treatment duration, as well as non-uniformity in follow-up interval and sample collection time [16].

Chemotherapy-related ovarian toxicity is a major concern for young cancer patients who receive chemotherapy with a curative intent. This may result in a decline in fertility potential on one end and menopause-associated comorbidities on the other. For patients who have not completed family, aspects on family planning should be discussed in advance especially in the light of increasing availability of effective means to preserve ovarian function and reproductive capabilities for patients who need various anti-cancer therapies.

It has been shown that over 30% of cancer survivors suffer from fatigue, anxiety, depression and impairment of physical functioning affecting their quality of life [48–49]. Studies on aspects concerning quality of life and comorbidities associated with chemotherapy-related menopause, including cardiovascular disease and osteoporosis, will enable patients as well as health care providers to better prepare patients for impacts of long-term sequelae of anti-cancer treatments.

## Supporting Information

S1 Table Clinical information database of study subjects. (XLSX)
